# Brexit, trade and the governance of non-communicable diseases: a research agenda

**DOI:** 10.1186/s12992-023-00956-3

**Published:** 2023-08-23

**Authors:** Benjamin Hawkins, Pepita Barlow, May CI van Schalkwyk, Chris Holden

**Affiliations:** 1 MRC Epidemiology Unity, University of Cambridge, Addenbrookes Hospital, Hills Road, Cambridge, CB2 0QQ UK; 2 Department of Health Policy, London School of Economics and Political Science, London, UK; 3 London School of Hygiene and Tropical Medicine, London, UK; 4 School for Business and Society, University of York, York, UK

**Keywords:** Brexit, Trade, Health, Non-communicable diseases, Industry, Policy-making, UK.

## Abstract

**Background:**

The UK’s post-Brexit trade strategy has potentially important implications for population health and equity. In particular, it will impact on the structural risk factors for non-communicable diseases (NCDs), including the consumption of health-harming commodities such as tobacco, alcohol and ultra-processed food and beverages. This article catalogues recent developments in UK trade policy. It then presents a narrative review of the existing research literature on trade and health and previous, prospective studies on the health impacts of Brexit. In so doing it identifies key questions and foci for a future research agenda on the implications of UK’s emerging trade regime for NCD prevention.

**Main text:**

We identify five key areas for future research. (1) Additional scholarship to document the health effects of key trade agreements negotiated by the UK government; (2) The implications of these agreements for policy-making to address health impacts, including the potential for legal challenges under dispute settlement mechanisms; (3) The strategic objectives being pursued by the UK government and the extent to which they support or undermine public health; (4) The process of trade policy-making, its openness to public health interests and actors and the impact of the political and ideological legacy of Brexit on outcomes; (5) The impact of the UK’s post-Brexit trade policy on partner countries and blocs and their cumulative impact on the global trade regime.

**Conclusions:**

Further research is urgently need to understand the ways in which the UK’s post-Brexit trade strategy will impact on NCDs and policy responses to address these, including the openness of the trade policy architecture to health issues. The outcomes of this process will have wider systemic effects on the global trade regime with implications for health. Researchers must be cognizant of the ideological components of the policy debate which have been absent from previous analysis of Brexit, trade and health.

## Introduction

The result of the 2016 referendum on membership of the European Union (EU) plunged the United Kingdom (UK) into its greatest political crisis since the end of the Second World War, precipitating a fundamental reorientation of its foreign and economic policies. While its supporters had offered little clarity about what Brexit meant in practical terms, the apparent benefits of an independent trade policy had featured heavily in the referendum campaign, and became a key objective for the UK government in the withdrawal negotiations. The decision to leave the EU’s customs union (CU) and common commercial policy posed a number of challenges. In the first instance, the UK would need to establish a new legal basis for its economic and political relationship with its closest neighbour and most important trading partner. In addition, the UK would need to replace more than 40 trade agreements with 70 countries from which it had formerly benefitted as an EU member-state. The UK’s exit from the EU in January 2020 occurred as the first cases of Covid-19 were identified in Europe and the negotiation of both subsequent Trade and Co-operation Agreement (TCA) with the EU and these ‘rollover’ agreements took place as the pandemic unfolded. Despite the capacity pressures this placed on governments across the continent the UK exited the post-Brexit transition phase as scheduled on 1 January 2021.

The UK government identified trade agreements with the United States of America (US), Australia, New Zealand and India as key strategic priorities – given their historical connections to the UK, and the absence of EU trade agreements with those countries – along with membership of the Comprehensive and Progressive Agreement for Trans-Pacific Partnership (CPTPP) as part of a wider strategic reorientation away from Europe and towards Asia.

The UK’s post-Brexit trade strategy has potentially important implications for population health and equity. To take a widely discussed example, the possible access of American healthcare providers to the UK’s National Health Service (NHS) as a condition of a UK-US trade agreement has been a source of great controversy due to fears that this will drive up healthcare costs and reduce quality and accessibility of care [1, 2]. In addition, international trade and investment agreements (TIAs) have significant (positive and negative) effects on the structural determinants of health and risk factors for non-communicable diseases (NCDs).

The latter represent one of the most significant health challenges facing the UK. NCDs are among the leading causes of death and disability in the UK for both males and females [3]. Risk factors for NCDs, namely poor diet, smoking, alcohol use and physical inactivity, represent leading drivers of the burden of premature death and preventable ill-health and widening of health inequities in the UK [4]. Compared to other European countries the UK experiences a greater prevalence of largely preventable NCDs including chronic obstructive pulmonary disease (COPD), asthma, obesity, and some types of cancer [5–7]. The UK population also has a greater intake of highly processed food compared to other European countries and, in 2018, less than 30% of adults in England consumed the recommended five portions of fruit and vegetables a day, with low intake concentrated among deprived communities [8–10].

Aggregate economic growth as well as the geographical and social distributions of wealth across society are impacted by TIAs, with implications for health inequalities. Agricultural and food production standards set out in TIAs significantly affect the quality and affordability of food and thus the population’s diet and nutritional profile. Many TIAs also confer rights and protections on transnational corporations (TNCs) to influence or challenge policies designed to protect or promote health – via regulatory co-operation or investor-state dispute settlement (ISDS) mechanisms – with important implications for the ability of national governments to enact policies to protect public health [11].

The content of such agreements, and thus their effects on health, are the result of political processes and power structures [12]. It is essential to explain the development of trade policy, and the effects of TIAs concluded, in terms of the political, economic and ideological context from which they emerge. The content and health effects of UK trade agreements have been, and will continue to be, shaped and mediated by the distinctive political economy of post-Brexit Britain.

This article reviews recent developments in the scope and politics of UK trade policy, and the existing research literature on trade policy, Brexit and health, in order to identify the key questions to be addressed by a future research agenda on the implications of the UK’s emerging trade regime for policies to address NCDs. In particular, it focuses on the ability to regulate structural risk factors relating to diet, nutrition and health-harming commodities such as tobacco, alcohol and ultra-processed food and beverages.

It begins by outlining the institutional architecture and governance of post-Brexit trade policy. Next, it surveys the research literature on trade policy and health globally, with a particular focus on recently emerging themes and insights. In addition, it summarises the more limited body of work on Brexit and health in the UK in order to highlight gaps in UK-specific research. The following section discusses the ideological and political factors shaping and constraining the development of post-Brexit trade policy and structuring the relationship between trade and health. The article concludes by outlining an agenda for future research to address the gaps identified in current scholarship. We adopt a political economy lens to explore critical questions for further examination. Identifying the political dynamics and power relations at play in such a fluid trade policy context is crucial also in identifying leverage points to influence such processes to promote health and equity in practice.

### The emerging post-Brexit trade regime

In the aftermath of the 2016 referendum, the direction of travel of the UK government’s policy towards the EU withdrawal negotiations quickly became clear. In her 2016 Conservative Party conference speech and, in greater detail, in her Lancaster House Speech of January 2017, Prime Minister Theresa May set out the UK’s negotiating position in terms of a series of ‘red lines’ that effectively precluded the UK’s membership of the EU internal market (IM) and CU [13]. This reflected the new orthodoxy amongst ‘leave’ supporters that the UK would only truly have left the EU, and honoured the outcome of the referendum, by leaving the IM and the CU. Furthermore, the UK’s ability to pursue an independent trade policy was portrayed as a unique opportunity to deliver greater choice and lower prices as part of their wider vision of a ‘global Britain.’

The decision to leave the IM and the CU had important implications for the UK economy. Exporters of agricultural products, manufactured goods and services would face new barriers to entry into the EU market. This was of particular significance for service providers, since it is in the area of services trade that the differences between the single market and a simple trade agreement is most obvious. The reliance of the UK on the services sector (representing around 80% of economic output and employment) and overseas trade (which accounted for 62.9% Gross Domestic Product in 2019) [14], meant that its exit from the IM, and the negotiation of a far looser trade agreement in the form of the TCA, would have significant economic consequences.

#### Post-Brexit trade governance

The decision to leave the CU necessitated the development of new political and administrative structures to govern the development and implementation of trade policy. The Department for International Trade (DIT) was created following the Brexit referendum in 2016, tasked with negotiating new TIAs, supporting British exporters, attracting foreign investment into the UK and overseeing stakeholder consultation processes to facilitate transparency and participation in trade negotiations. At the same time, the House of Commons International Trade Committee (ITC) was formed, taking on the task of examining the activities of the DIT and its associated public bodies [15]. In February 2023, DIT was merged with the Department for Business, Energy and Industrial Strategy to form the new Department for Business and Trade and the formation of a new House of Commons Business and Trade Select Committee to oversee the ministry’s work. Disbanding the ITC meant a reduction in the level of parliamentary scrutiny over trade issues given the wider policy remit of its successor.

Having left the EU, the UK now sits as an independent member-state of the World Trade Organization (WTO) with its own schedule of commitments (i.e. the tariff rates, quotas and other conditions it offers to importers). Policies implemented by the UK that are seen to be trade diverting or discriminatory may, therefore, be subject to challenge under the WTO by other members, including now the EU. This is especially likely where the UK may seek to introduce novel policies that differ from other large nations or blocs. The first such dispute was initiated by the EU against a UK scheme to support the development of low-carbon electricity in March 2022, and was resolved in July that year following arbitration [16]. While UK policies could have been challenged before the WTO when the UK was an EU member-state, the power dynamics are very different as an independent trading nation. Not only has the UK lost the collective support and representation of the EU at the WTO – which may deter cases being brought by states dependent on access to the much larger EU market – but it faces the increasing prospect of the EU, as a powerful, geographically-proximate economic bloc with significant exposure to the UK economy, policing its trade policy.

#### Ongoing negotiations

The TCA with the EU was concluded in late December 2020 and entered into effect on 1 January 2021 at the end of the post-Brexit transition period. An agreement with European Economic Area (EEA) members, Norway, Liechtenstein and Iceland, was announced in June 2021, which entered into force in January 2022. At the time of writing, the UK had successfully negotiated the continuation of a further 36 existing agreements covering trade relationships with 67 countries to which it was party as an EU member-state. These were agreed on similar or slightly amended terms to the *status quo ante*.

In June 2021, the UK agreed the terms of a trade agreement with Australia (signed 15 December 2021; in effect from 31 December 2022) and announced the following October that a similar agreement had been reached in principle with New Zealand (signed February 2022; in effect from 31 May 2023), reflecting Brexiters’ emphasis on the ‘Anglosphere’ as an alternative economic and diplomatic context for the UK to exist outside of the EU [17]. A digital trade agreement was also concluded with Singapore in February 2022, entering into force the following June. In February 2021, the UK formally applied to join the CPTPP; a move reflective of a wider strategic reorientation of the UK towards the Asia-Pacific region set out in the *Integrated Review of Security, Defence, Development and Foreign Policy* [18]. In March 2023 it was announced that UK had successfully concluded negotiations on CPTPP accession and it formally joining the bloc in July 2023.

Despite the key strategic importance placed on this by Brexit advocates, trade negotiations between the US and the UK have been repeatedly delayed. However, a mutual recognition agreement between the two countries – under which each party accepts conformity assessment of goods undertaken by the other – was signed in February 2019. In September 2022, the UK government was forced to concede that a full trade agreement remained a distant possibility given the differences in political orientations and priorities between the parties. In October 2022, reports emerged that negotiations on a UK –India trade agreement had also stalled over the proposed mobility chapters in the agreement and the issuing of visas for Indian citizens to work in the UK.

### Understanding the relationship between trade and health

There is now an extensive literature on the relationship between trade and health [12], reflecting the proliferation of TIAs concluded since the turn of the millennium and the increasing recognition of their societal and environment impacts [19]. We undertook a purposive literature review of studies relating to our principle concern with NCD risk factors and prevention policies and supplemented this with additional sources identified from references within the initial sources sampled [20]. In addition, we sought to identify studies that had engaged specifically with the issue of Brexit and the direct and indirect consequences of the post-Brexit trade regime for population health. It is possible to identify several closely related and, at times, overlapping strands in this literature, which we discuss in turn below.

#### The impact of trade on health and nutrition

One of the most developed strands of literature on trade and health focuses on the implications of TIAs for food and beverage consumption and nutrition, particularly in low and middle income (LMIC) settings [21–27]. For example, abolishing tariffs on products high in sugar, salt and saturated fat is associated with increased sales of these products in some contexts and there is evidence that lower tariffs on unhealthy food are associated with obesity. For example, Giuntella et al. [28] find that exposure to food imports from the U.S. explains about 10% of the rise in obesity prevalence among Mexican women between 1988 and 2012. Boysen and colleagues [29] also modelled the links between tariffs on highly processed foods relative to unprocessed foods and the prevalence of both obesity and underweight among adults. They found that in middle-income countries in sub-Saharan Africa, a 1% increase in the tariff differential between processed and unprocessed foods corresponded to a 0.18% decrease in obesity prevalence. This points to the important role of tariffs in setting the relative prices of highly processed and unprocessed foods – an important driver of excess consumption of these products at the population level and across different socio-economic groups. Friel et al [30] further identify how restrictions on domestic support (e.g. agricultural subsidies) have important effects on domestic food production while ‘behind the border’ measures designed to ensure common standards for food production (to ensure fair competition between actors) may reduce the regulatory space available to governments to take counteracting measures [see also 31].

Recent scholarship on TIAs and nutrition emphasises the political nature of trade policy and the choices made by governments when entering into such agreements. For example, Friel and Jamieson [32] argue that poor diet and associated health outcomes are the result of a political and economic context shaped (in significant part) by trade patterns and the nexus of agreements governing these. The content of these agreements reflects, in turn, the ability of powerful economic actors, with privileged access to trade negotiations, to present their particular interests as being in alignment with governments’ objective to promote economic prosperity via market liberalisation. While these findings relate principally to the role of TIAs in fostering changing diets in LMIC contexts, they highlight issues of relevance to the UK. These include concerns about access to domestic food markets by international suppliers, the ability to support domestic agriculture and food producers, food production standards, the relative affordability of healthy and unhealthy foods and the nutritional content of population diets.

#### International trade, policy space and the right to regulate

A second strand in the literature identifies the ways in which TIAs, and the wider global trade regime, can restrict the ability of national governments to enact health-protective measures. Dür et al. [19] find that ‘behind the border’ measures to reduce non-tariff barriers to trade are especially important to trade facilitation, meaning deeper agreements have greater effects on trade volume. Yet these may reduce the policy space open to governments to adopt product regulations such as those designed to reduce consumption of health-harming products or environmental factors impacting health. Thow and McGrady [33] identify inherent tensions between governmental measures to incentivise and attract foreign direct investment and upstream policy measures designed to limit the production, sale, marketing and consumption of processed food and drink products, which run counter to the pro-business policies designed to attract investors.

In addition, TIAs may contain regulatory co-operation and ISDS mechanisms that can be used to shape the content of, or challenge, health policies. The former require governments to inform trade partners about any new laws that may affect commitments within a TIA and offers affected parties an opportunity to comment on, and thereby shape, the policy process. The latter, meanwhile, allow private corporations, in their capacity as investors, to challenge laws and other regulatory measures adopted by national (and, in devolved and federal systems, sub-national) governments if they are felt to infringe any of the rights guaranteed by these agreements. Hawkins and Holden [34] employ the concepts of ‘veto points’ and ‘venue shopping’ to theorise the ways in which TIAs may create additional barriers to policy change. They allow TNCs to exploit multiple policy-making processes and contexts, especially in areas in which the measures adopted run counter to the logics of commerce and consumption, which underpin the global trade and investment regime [see also 25, 26]. The potential for legal challenges under ISDS mechanisms may mean governments self-censor their legislative agenda, avoiding or watering down potentially controversial measures and leading to a so-called ‘chilling effect’ on health policies. This was evident in the case of tobacco industry attempts to resist regulation of product packaging and labelling in multiple contexts [35–37]. The lack of a cumulative jurisprudence in TIA law adds to uncertainty for governments. In other words, ISDS challenges can be brought even where previous judgements in similar cases may have found in favour of those adopting a particular public health policy, thus increasing the disincentives for governments to adopt controversial policies. The overall effect is to create uncertainty and inertia on the introduction of a progressive measure not just in public health but in other areas such as environmental protection [38–40].

Challenges to health regulations can also arise in other contexts such as the WTO’s Technical Barriers to Trade Committee. WTO members agree to follow rules set out in a suite of agreements, including those in the WTO’s Technical Barriers to Trade (TBT) Agreement, which seeks to minimize ‘unnecessary’ trade costs that are created by regulatory differences between states [41]. According to the agreement, governments are required to submit any policy that may impact trade to scrutiny by WTO members. Other members can then challenge a policy that they deem to be inconsistent with the TBT Agreement, for example because the regulation creates trade costs that are considered to be higher than necessary to meet the regulation’s objectives. Between 1995, and 2016, WTO members raised challenges to 250 health policies and regulations at meetings of the TBT Committee, including front-of-pack nutrition labelling policies and alcohol health warning labels [42]. Analyses of these challenges suggest that they are used by industry actors to contest policies affecting their products, as the main arguments raised are closely aligned with key arguments made by industry groups in other fora [43].

More recent scholarship emphasises the different forms of regulatory chill and potential mitigation strategies that governments may adopt. For example, Schram et al. [44, 45] identify three forms of regulatory chill – anticipatory, response and precedential chill – in the context of nutrition and alcohol policy in South Africa [see also 45]. Tienhaara [46], meanwhile, highlights the difficulty of detecting and measuring regulatory chill and thus in fully assessing its significance for policy development (or non-development)[46]. Garton et al. [26] distinguished between the ‘substantive,’ ‘structural’ and ‘procedural’ ways in which the domestic policy space may be constricted, with the latter (relating to the process of policy-making) being the biggest threat to regulatory capacity. Local factors – including policy actors, institutions and political context – are essential for determining policy outcomes. They conclude that sufficient policy space exists for well-designed health protection policies to be compatible with commitments under trade agreements, highlighting that many threats by TNCs are not inevitably grounded on robust legal arguments. Similarly, Dorlach and Mertenskotter [47] argue that governments can ward against the threat of challenge by structuring and framing policies in ways designed to insulate them from such disputes. Garton et al. [25], meanwhile, argue that policy-makers may overestimate the threat of policies being challenged under WTO law or TIAs, and their chances of losing any case brought against them. They reiterate that the framing of specific policies is key to justifying their necessity, and thus their compatibility with trade laws and the obligations they confer on governments.

Others too have examined the ways in which trade agreements may be designed in ways to protect the ‘right to regulate.’ Janardhan [48] discusses the implication of trade agreements for alcohol policy, suggesting the potential for health ‘carve- outs’ as a means of protecting this and other policy areas. Such measures were proposed for tobacco products only in the initial negotiations for the CPTPP and were included in a weakened form in the agreement [49]. Jarman [50], meanwhile, discusses the effectiveness of tobacco industry ‘denormalisation’ as a tobacco control strategy (e.g. the progressive exclusion of tobacco industry actors from health policy-making via the Framework Convention on Tobacco Control [FCTC]) and the potential challenges to these measures under TIAs on the basis of principles such as fair and equitable treatment, given past cases brought by tobacco companies against Australia and Uruguay. A government, if it is to be successful in defending and legitimising the exclusion of an entire industry, must demonstrate its own competence and adherence to robust systems of governance. Stable and predictable systems of governance are also essential for enabling a government to prepare for and weather the legal challenges that may be mounted against it by those opposed to certain public health measures.

These studies reiterate the potential threat posed by TIAs to both the right and willingness of governments to adopt health and other social policies and the importance of policy design in protecting regulatory space for these. This underlines the importance of understanding the processes through which trade and health policy are developed, the actors to which policy-making processes are open and the interests that they reflect. From a public health perspective, attention needs to be paid also to the ways in which the norms and assumptions underpinning current trade regimes can be challenged, to promote health interests and diminish the influence of health-harming industries over the trade policy space.

#### The politics of trade policy and its openness to health

A third strand in the literature looks at the formulation of national trade policies, and the ideological assumptions, issue framings and institutional structures that render trade policy formation amenable to industry versus public health influence. Turning first to the ideological assumptions underpinning trade policy processes, Townsend et al. [51] analyse submissions to the Australian government during negotiations for the Trans-Pacific Partnership Agreement (the predecessor to the CPTPP). They found that industry actors typically adopt a ‘neoliberal’ framing of trade, venerating free enterprise, and, therefore, have more discursive power to influence the trade agenda. Similarly, Baker et al. [52] identify what they term a ‘productivist’ paradigm dominating the trade policy space, both in Australia and negotiating partners, which emphasised economic growth above other outcomes, permitted few opportunities for civil society engagement and marginalised discussion of the links between trade policy and nutrition. Friel et al. [53] conclude similarly that trade policy is dominated by a narrowly conceived form of ‘realpolitik’ framed in terms of maximising exports and growth, from which health and equity considerations are excluded.

Scholars seeking to challenge this narrow, reductionist conception of trade policy have employed the concept of ‘policy coherence’ to highlight the inter-connectedness of trade and other areas of public policy; and the need for trade policy to reflect the wider responsibilities of governments in areas such as public health [see also 53–56]. Yet the idea of coherence can be of limited utility in advocating for health-inclusive trade policies since it assumes a genuine commitment to public health by governments beyond trade. It is possible that governments’ approach to trade is perfectly coherent with their overall policy orientation if this relegates health below other (often economic) outcomes across the policy spectrum. What is required instead is to critique governments from a more explicitly normative position, designed to promote health as an objective in trade policy and as a cross-governmental priority.

Schram [56] explains the marginalization of public health issues in the context of trade and investment negotiations in terms of the obvious resource imbalances between commercial and health actors and the predominance of neo-liberal ideology. This is evident in the increasing marketisation of the state, the economisation and individualisation of public policy, and the narrow delineation of the trade policy community [see also 51, 55, 57]. Consequently, public health actors are not seen as legitimate trade policy actors, resulting in their effective exclusion from both trade policy formulation and negotiations. Milsom et al. [58], meanwhile, identify three aspects of corporate power – instrumental, structural and discursive – to explain how harmful commodity industries use TIAs to encourage ‘non-decisions’ or inaction in public health policy-making [see also 59–61]. They argue that the ideological embeddedness of pro-industry agendas in the trade policy space privileges economic actors and marginalisation of health interests, leading to imbalances in access to policy-makers and policy outcomes.

Finally, O’Brien et al. [62] examine the interconnections and path dependency between trade agreements. They highlight how the adoption of particular standards within a regional trade agreement may influence or structure regulatory approaches adopted in subsequent bilateral agreements, through the example of alcohol labelling. The use of supplementary labels for health information (versus the mandatory inclusion of this on standard product labels) within the CPTPP led to the uptake of similar measures in subsequent agreements (e.g. Singapore-Australia Free Trade Agreement). The structuring effect of the CPTPP on trade negotiations in the region poses a significant, but not insurmountable, challenge for governments wanting to introduce stricter labelling requirements [63].

These studies reflect not just the importance of studying trade policy processes in specific settings, but of understanding the wider political and ideological contexts in which these are developed and pursued by governments. Given the interconnectedness of different regional and bilateral agreements, and the development of specific trade norms and standards within regional blocs, it is important to examine the ways in which specific TIAs entered into by countries may facilitate or preclude future agreements with other parties, or create precedents for the content of subsequent agreements into which that country may enter.

#### The impacts of Brexit on trade and health

A related literature examines the specific implications of Brexit for health and trade, focussing principally on the potential health effects of the UK’s future relationship with the EU and the processes through which interests are articulated and trade policy is made. These analyses are largely prospective, modelling different scenarios for the UK’s future economic relationship with the EU, and do not engage with the implications of trade for NCDs. For example, Dalingwater [64] discusses the choices facing the UK government in setting its economic and commercial policies in terms of the concept of sovereignty, identifying the implications for agricultural and food production standards in a potential trade agreement between the UK and the United States of America (US) as posing a particular threat to public health. Similarly, accession to the CPTPP would signify a shift towards the US regulatory model, creating potential divergence with EU standards and thus barriers to any future deepening of trade relationships with the EU.

Modelling by Freund and Springmann [65]found that a ‘hard’ Brexit (as negotiated in the WA and TCA) would have significant negative impacts on diet, nutrition and associated health outcomes. This would lead to a net increase in diet-related mortality as costs for healthy, imported foods increase, but could potentially be offset by wider tariff reductions. They predicted a UK-US TIA could lead to a tripling of the negative health impacts of Brexit due to an influx of cheaper energy-dense, nutritionally poor foods [65]. More recently, analysis by the Centre for Economic Performance at the London School of Economics has identified Brexit as a significant driver of inflation, accounting for around 30% of food price increases [66, 67]. The delayed introduction of additional border checks on imports into the UK are predicted to create additional inflationary pressures on the UK’s import-dependent food sector [68].

Dayan et al. [69, 70] identify considerable potential effects of leaving the single market on the health and food systems and identified a lack of an overall strategy for governing aspects of health policy previously under EU competency, including those falling within the remit of the devolved administrations [see also 71]. They identified a policy process shrouded in secrecy, with poor data sharing and limited engagement with stakeholders beyond key commercial actors such as the pharmaceutical industry. This resulted in limited oversight and accountability of government actions by parliament.

Similarly, van Schalkwyk et al. [15] examine UK post-Brexit trade governance and the activities of the DIT and the House of Commons ITC formed after the 2016 referendum. They identify serious weaknesses in trade governance and procedures with important implications for population health and social justice. Trade policy processes, they find, are characterised by a lack of openness, transparency, participation, integrity and accountability, even to parliament. DIT, for example, failed to engage, or even communicate effectively, with key policy actors and stakeholders, including the devolved administrations. In keeping with studies in other contexts (cited above), they find that the health and equity implications of trade policy, to the extent they were discussed at all, remained a low-salience issue and its scope narrowly defined (i.e. in terms of specific issues such as food safety and the NHS versus population health). Similarly, Fahy et al. [72, 73], examining the UK government’s position during the TCA negotiations, identify a lack of openness and oversight in the trade policy process and insufficient engagement with actors to identify and potentially mitigate the negative consequences of the agreement (and wider trade policy) for population health.

Finally, Siles-Brugge [74] highlights the importance of recognising the emotive dimension of trade politics. He analyses competing visions of ‘soft’ and ‘hard’ Brexit within the Westminster polity, and their respective appeals to ‘technocratic’ and ‘emotive’ spatial imaginaries. While the former emphasises economic rationalities and gravity models of trade as the justification for close alignment with the EU, the latter succeeded through focussed on emotionally resonant, historical-cultural ties with the ‘Anglosphere’ as the imaginary locus of the post-Brexit UK [see also 75]. This builds on findings from the wider trade and health literature on the importance of issue framing, adding an important, affective component to our understanding of trade policy dynamics.

#### Key insights for post-Brexit trade and NCDs

The preceding literature review identifies key themes that are of relevance when considering the implications of the UK’s emerging, post-Brexit trade regime for NCDs, their inequitable distribution, and future research on this topic. Firstly, previous studies identify how TIAs have significant impacts on NCD risk factors, including the food system and population nutrition. TIAs impact agricultural production standards and the availability, and relative affordability, of healthy and health-harming foods. This is of particular relevance to the UK given the high proportion of fruit and vegetables that are imported into the UK. The intersection of the Covid 19 pandemic, the global energy crisis following Russia’s invasion of Ukraine in February 2022 and the additional import restrictions as a result of Brexit have had significant inflationary pressure on essential commodities including food, with implications for diet, nutrition and associated public health [66]. However, it should be noted that much of the existing literature on the effects of TIAs on diet, nutrition and health focuses on LMIC settings with particular and distinct health challenges and policy-making environments. The UK, meanwhile, is a high-income, post-industrial economy, heavily reliant on the service sector and international trade, with currently high levels of protection for human and animal welfare and the environment. Thus, while these findings are of relevance to the UK, the specific effects of the post-Brexit trade regime on food supply and population nutrition will differ in important ways.

Secondly, TIAs may shrink the policy space open to national governments. They include regulatory cooperation and ISDS mechanisms, which can prevent the adoption of, or otherwise weaken, health-protective policies and/or lead to their challenge once enacted. The proliferation of these agreements is likely to affect the ability of the UK and devolved governments to regulate to protect health. Similarly, as an independent member-state, the UK is now open to potential challenges to its trade policies at the WTO, including by the EU. However, as the studies discussed above suggest that it is possible to design and ‘frame’ health-protective policies in ways that reduce the likelihood of challenges both at the WTO and via ISDS mechanisms under TIAs.

Thirdly, important issues arise about the governance and formulation of trade policy, the level of transparency and accountability of the policy-making architecture, and its openness to public health versus the commercial sector. Health issues appear to be of low salience and given insufficient consideration in trade negotiations despite the widespread effects that TIAs can have on population health. Furthermore, health actors are not seen to be part of the trade policy community and do not enjoy significant access to policy-makers. In contrast, commercial actors, including those in health-harming industries, are afforded significant opportunities to shape the trade policy agenda. The exclusion of health (and other social issues) from the trade policy arena reflects the narrow ‘productivist’ lens through which trade is viewed in trade ministries and the wider epistemic community and the role of neo-liberal ideologies in reproducing the *status quo*. Even where health actors do have opportunities to provide input on trade negotiations (e.g. via consultations), they are less persuasive to policy-makers imbued with deeply embedded, neo-liberal perceptions of trade. This underscores the importance of ideological alignment and misalignment, as well as the role of framing and affect, in shaping how health issues are taken into account in policy debates.

In terms of the UK specifically, existing studies identify a lack of openness by DIT towards policy actors in civil society, the devolved administrations and other parts of central government evident in the wider literature. Those processes that have been put in place to facilitate transparency and participation have important weaknesses, limiting the extent to which external stakeholders can scrutinise trade negotiations or provide meaningful input.

### The political-economy and ideology of the post-Brexit trade regime

The existing literature on trade and health offers important insights and points towards potential research foci for scholars seeking to understand the relevance of the UK’s emerging trade policy for NCD prevention and wider health policy. However, post-Brexit trade policy cannot be separated from the politics of Brexit [17], and longer-term Eurosceptic movements [76, 77], which continue to shape the UK’s political landscape. The UK is confronting one of the most fundamental reorientations of economic and diplomatic policy ever undertaken by an advanced economy, while navigating the political consequences of the referendum that precipitated this. In this state of flux, in which the economic and political direction over the coming decades may be set, trade policy is significant in at least three ways.

Firstly, trade policy is at the heart of debates over the success of Brexit and the long-term sustainability of the UK’s current geopolitical status. The 2016 vote was framed as a unique opportunity for the UK, and its people, to ‘take back control’ [17]. A key focus of this narrative was the ability of the UK to conclude its own trade agreements and the prosperity and self-confidence this would bring as it retook its seat at the ‘top table’ of international organisations such as the WTO [17]. Consequently, the success of the UK’s trade policy became a touchstone for evaluating the success of Brexit more generally. While the approach to trade negotiations has shifted under Prime Minster, Rishi Sunak, his immediate predecessors – under whom the majority of the UK’s post-Brexit agreements were negotiated – presented these deals as a vindication of the model of Brexit they supported. In this context, each new agreement has a vital symbolic currency within these debates, independently of their economic impact. They are counted as evidence that the UK has not lost out on prior agreements by leaving the EU, or has gained something by closing deals where the EU has none. At the same time, they symbolise the performative de-anchoring of ‘global Britain’ from the European mainland as it set its sights on the more dynamic and faster growing markets of Asia and its natural home in the ‘Anglosphere’ [78]. Yet the symbolic importance of the TIAs concluded to date far outweighs their actual economic effects. The simple numbers game of how many agreements had been rolled over or newly struck was foregrounded, in public discourse at least, over evaluation of their economic impact, and the extent to which they offset the loss of access to, and additional frictions in accessing, the EU market under the TCA versus as a member-state.

Secondly, the ability of the UK to pursue an independent trade policy is inextricably linked to a wider, deregulatory political agenda. For many Brexit supporters, the act of leaving the EU was not an end in itself, but an important and necessary step in a longer-term political project to curtail the allegedly overbearing nature of the modern state and its implications for individual freedom and responsibility. Within this group, Euroscepticism overlaps with a fundamental rejection of state regulation and the types of public health policies designed to tackle NCDs – such as tax and other price increases on health-harming products – that they dismissively caricature as the ‘nanny state.’ This coalition of anti-EU, anti-regulatory and anti-public health discourses is evident in organisations such as the Institute of Economic Affairs (IEA) and their various outputs and in the policy platform of the current Conservative government. Brexit was a necessary, but not sufficient, condition for a fundamental reorientation of the UK’s social and economic model. Leaving the constitutional and legal constraints of the EU opens the possibility for the fundamental deregulation of the UK economy, and the rolling back of associated rights and protections, for example in food productions standards. At the same time, these policies can be facilitated, or further embedded, by the UK’s independent trade policy and the provisions of TIAs entered into by the UK government. While such policies may be unpopular with the electorate, these agreements provide political cover for their sponsors. They can be sold to the public under the guise of economic necessity; as the price of doing business in new markets, which will be offset by the putative benefits for UK exporters and the economic growth they will bring.

Thirdly, and relatedly, trade agreements with other regional blocs (i.e. CPTPP), or countries with different regulatory models to the EU, serve to lock in the new economic model that they favour, and create important practical and legal barriers to any attempt by a future UK government to rejoin the EU or to remain within the wider EU orbit. The US in particular represents an alternative, and largely incompatible, regulatory model to that of the EU, as is evident in the now frozen negotiations of the proposed Trans-Atlantic Trade and Investment Partnership (TTIP) [79]. To take a widely discussed example, the lower agricultural production standards – which necessitate the chlorine treatment of poultry to ensure its safety – mean that meat products originating in the US cannot be sold on the EU market. Any subsequent UK-US TIA which allowed US meat and poultry into the UK market – and which forbade labelling of its origin, as may be the case under non-discrimination clauses common in such agreements – would pose significant barriers to the re-entry of the UK into the single market via the EEA, not to mention significant problems for the functioning of the Northern Ireland protocol to the withdrawal agreement, which seeks to ensure an open border between the UK and the EU on the island of Ireland. Despite the political motivation for performative and material divergence from the EU, developments within EU trade policy will continue to impact on the UK due to its geographical proximity and the size and importance of its market to the UK. As such, analyses of UK trade policy must continue to monitor developments across the channel and their implications for trade and health.

At the same time the, UK’s conclusion of bilateral trade agreements with third countries, and its accession to regional blocs such as CPTPP, will have important impacts on partner countries and wider systemic effects on regional and even global trade regimes. For example, concessions given to Australian agricultural producers in the UK-Australia trade agreement further complicated the negotiations for the UK to acceded to the CPTPP as member countries demanded similar terms under the principle of most-favoured nation. This is in keeping with studies of the path dependency of intra-regional trade agreements identified above [62, 63], and may have longer term effects on the development of the bloc as new members shift the dynamics of inter-state bargaining. Relatedly, the terms of agreements concluded by the UK with low and middle-income countries may have important implications for health and development in those countries and their achievement of the UN Sustainable Development Goals.

In summary, UK trade policy is of clear and obvious relevance for public health and the control of NCDs, both in terms of its direct consequences for health policy and its importance within wider debates about the economic and political direction of post-Brexit Britain. At the same time it is vital to see trade policy not simply in economic or transactional terms, but to understand its wider symbolic importance within the politics of Brexit. The scale and speed of these changes reveal the need for further research across these inter-related spheres to support health-promotion.

### Key objectives for a new research agenda

The previous literature on trade and health, and the more limited set of studies on the implications of Brexit for public health, raise a number of issues relating to the implications of the post-Brexit trade regime for NCDs, which require additional scholarly attention. However, any research agenda needs to be sensitive also to the particularities of the UK context. Firstly, additional scholarship is needed to understand and document the health effects of key trade agreements concluded by the UK government (i.e. with the EU, Australia and New Zealand) or currently under negotiation (i.e. with the US and for accession to the CPTPP) and their implications for NCDs and NCD prevention policies. This requires a careful mapping of their impacts on the agricultural system and food supply chains; the nutritional profile and accessibility of health-sustaining foods such as fruits and vegetables; and the availability, affordability and marketing of health-harming products. This will build on previous studies that have attempted to model the effects of different types of Brexit and the UK’s relationship with the EU. At the same time, it will contribute to a more nuanced debate around the effects of the post-Brexit trade regime on health and the wider economy, which seeks to move beyond the simple ‘numbers game’ of counting new agreements.

Secondly, a new research agenda must examine the ability of the UK government, and those in the devolved administrations, to regulate effectively to address health impacts while minimising the potential uses of regulatory co-operation and ISDS chapters within TIAs by industry actors, as well as the WTO dispute resolution mechanisms, to prevent policy measures that commercial actors perceive to harm their interests.

Thirdly, greater insights are needed into the strategic objectives being pursued by the UK government in terms of trade policy, and the extent to which they support or undermine public health. What are the key economic and geo-political priorities underpinning the UK trade strategy? How is the UK government seeking to secure its aims and objectives? What is the underlying rationale for the trade strategy being pursued by the UK government? The instability in UK politics in the post-Brexit era – exemplified by a period of seven weeks in the autumn of 2022 which saw three different Conservative Prime Ministers in Downing Street – has implications also for the specific trade objectives pursued by the UK Government. While the administrations of Boris Johnson and Liz Truss had prioritised the rapid conclusion of agreements with commonwealth partners, taking an antagonistic approach to the EU over the NI protocol, the premiership of Rishi Sunak appears to have signalled a more pragmatic approach to trade policy, most visible in the de-escalation of tensions with the EU. The agreement of the ‘Windsor Framework’ in February 2023 signalled a shift in focus from the UK government from collecting and counting new trade agreements to ensuring the effective implementation of those they have in place or will soon conclude. With the UK facing a general election and a possible change of government by January 2025 at the latest, additional research will be needed to monitor the trajectory of UK trade strategy under a future Labour, or newly-returned Conservative, government.

Fourthly, and relatedly, we need a more nuanced understanding of the formulation and governance of trade policy, and the extent to which the trade policy architecture is open to, and can be shaped by, different actors. To what extent does the UK’s strategy reflect the interests and objectives of key economic, policy and civil society actors? Is health seen as a trade issue and are health actors taken to be a legitimate part of the trade policy community? Similarly, are health issues and actors included in trade policy deliberations and, if so, through what mechanisms and at which stages of the policy process? How do health actors seek to gain access to decision-makers and to articulate their political priorities? How do their experiences compare to those of other actors and interest groups such as those within the commercial sector? How has this changed with the disbanding of DIT and the formation of the new Department for Business and Trade? The recent experience of the Covid 19 pandemic and the current cost of living crisis – in which the intricate connection between population health and economic well-being have come to the fore – may offer potential opportunities for health actors to integrate themselves more centrally into the development of economic and commercial policy.

This research must pay particular attention to the political, economic and ideological context in which UK trade policy is being developed. Trade policy occupies a totemic position within the politics of Brexit. It is inextricably linked to the idea of an independent, ‘global’ Britain and its relationship with both Europe and the alternative economic and political contexts of Asia and the Anglosphere. Consequently, we need to understand the ways in which the politics of Brexit are shaping the UK’s trade strategy, the timing and content of the negotiations pursued and the agreements concluded by the UK government. This was evident in the recent claims by former trade minister, George Eustice [80], that the timing of the UK-Australia trade agreement, and thus the significant concession made by the UK to ensure this, had been driven by political expediency, resulting in a sub-optimal outcome for the UK economy. Future research must, therefore, seek to understand the ways in which the political context affects the openness of the trade policy agenda to health issues, and how health advocates frame their arguments to appeal to the government’s wider policy agenda.

Finally, further studies are needed of the external effects of UK trade policy. How will agreements concluded with third countries effects trade flows, wealth distribution and health outcomes in these. How will the accession of the UK to trade blocs such as the CPTPP shift the internal political dynamics and trade flows within these and with what effects on population health?

## Conclusions

The future direction of the UK’s emerging, independent trade policy, and its implications for NCD policies domestically and the global trade regime, remain unclear. Previous studies indicate that TIAs and global trade law have significant impacts on health, including NCDs, and the capacity of governments to make effective health policy. The existing literature on trade and health offers a guide to what the effects of the UK’s emerging trade policy may be for population health. Yet the specific effects of trade agreements depend on the particular positions that countries occupy within the global economy and the domestic political factors that shape trade policy. Understanding the direction of the UK’s post-Brexit trade regime, its implications for health and the potential for health actors to shape the policy-making process, thus requires an understanding of the very specific political context in which these processes are occurring, including the politics of trade within the political-economy of Brexit. In light of this, we have set out the contours of a new research agenda on the implications of the post-Brexit trade regime for NCDs and NCD policy in the UK.

## Data Availability

All data sources are referenced and in the public domain.

## Abbreviations

CPTPP: Comprehensive and Progressive Agreement for Trans-Pacific Partnership
CU: Customs Union
DIT: Department for International Trade
EEA: European Economic Area
EU: European Union
FCTC: Framework Convention on Tobacco Control
IEA: Institute of Economic Affairs
IM: Internal Market
ISDS: Investor-State Dispute Settlement Mechanisms
ITC: International Trade Committee of the House of Commons
LMIC: Low and Middle Income Country
NCD: Non-Communicable Diseases
NHS: National Health Service
TBT: Technical Barriers to Trade
TCA: Trade and Co-operation Agreement
TIA: Trade and Investment Agreements
TNC: Trans-national Corporations
TTIP: Trans-Atlantic Trade and Investment Partnership
UK: United Kingdom
US: United States of America
WTO: World Trade Organization
